# Regulatory Effects of Reward Anticipation and Target on Attention Processing of Emotional Stimulation

**DOI:** 10.3389/fpsyg.2020.01170

**Published:** 2020-06-03

**Authors:** Yujia Yao, Yuyang Xuan, Ruirui Wu, Biao Sang

**Affiliations:** ^1^Zhejiang University of Technology, Hangzhou, China; ^2^Shanghai Academy of Educational Sciences, Shanghai, China; ^3^East China Normal University, Shanghai, China

**Keywords:** target, reward anticipation, emotion processing, cue-target paradigm, eye tracking

## Abstract

Studies suggest that reward and emotion are interdependent. However, there are discrepancies regarding the interaction between these variables. Some researchers speculate that the inconsistent findings may be due to different targets being used. Although reward and emotion both affect attention, it is not clear whether their impacts are independent. This study examined the impact of reward anticipation on emotion processing for different targets. A cue-target paradigm was used, and behavior and eye-tracking data were recorded in an emotion or sex recognition task under the conditions of reward and non-reward anticipation. The results showed that when the target was related to the emotional attribute of the stimulus, the reward promoted the processing target information, thereby generating reward-oriented attention. When the target was unrelated to the emotional attributes of the stimulus, the reward did not promote the processing target information, and at the same time, individuals had negative emotional biases toward the emotional faces. The results revealed that, in addition to affecting the attention to emotional faces independently, the target regulated the promotion of reward anticipation to emotional attention and attention bias toward negative stimuli.

## Introduction

In the process of socialization, reward is often associated with positive emotions such as pleasure and satisfaction. Both reward and emotion have affective significance, defined as either negative or positive value to the organism (Pessoa, 2009). The relationship between reward and emotion has attracted the interest of researchers. Existing studies suggest that reward and emotion impact each other. Reward induces positive emotions (Berridge and Robinson, 2003). The reward circuit in the human brain is activated when an individual imagines a pleasant scene while reading a story (Costa et al., 2010) or stares at a photograph of a lover (Aron et al., 2005). Conversely, emotion, especially negative emotion, has an impact on reward. Chen (2013) reported that depression affects the reward system: the activation of reward-related brain areas decreases when depressed patients engage in reward processing. Similarly, during a gambling task, the activation of the reward circuit is lower in college students with high depressive symptoms than in those without such symptoms (Wei, 2015).

However, there are some discrepancies among previous studies on the interaction between reward and emotion. Some studies conclude that there is no interaction between reward and emotion. For instance, Murray (2007) noted that the neural substrates for emotion and reward were partially non-overlapping. The researcher noted a distinction between reward processing and emotional reactions, with the amygdala playing a crucial role in the latter and only a conditional role in the former. In another study, the cue-target paradigm, in which subjects were asked to attend to valid and ignore invalid spatial cues and motivation was manipulated by varying the magnitude and valence of a monetary incentive expected by the subjects for performing well on the task (Engelmann and Pessoa, 2007), was used to examine the impact of different reward conditions on the identification of vocabulary attributes of the target, and no interaction was found between reward and emotion (Kaltwasser et al., 2013). However, other researchers consider that there may be an interaction between them. Wittmann et al. (2008) found that reward strengthened memory only in the context of positive emotion. The same cue-target paradigm was used to investigate the effect of reward on emotional face recognition. A significant interaction was found between reward and emotion, and only negative emotion processing and bias effects were regulated by reward (Wei et al., 2014). Researchers speculate that the key reason for the divergence in the above findings may be the different targets. The target in the research of Kaltwasser et al. (2013) was independent of the emotional valence of the material (judging whether the target was concrete or abstract), while the target in the research of Wei et al. (2014) was related to emotional valence (judging whether the target was positive or negative). Wei and Kang (2014) demonstrated that when emotion was associated with the target, the reward effect of an emotional face (the difference in reaction times <*RTs*> between the non-reward condition and the reward condition) was greater than that of a neutral face. When emotion was irrelevant to the target, this effect did not exist. However, due to the limited information provided by RTs (Armstrong and Olatunji, 2012), this speculation needs to be further validated at other levels (e.g., attention) by other technologies (e.g., eye movement).

Both reward (Carmona et al., 2012) and emotion (Armstrong and Olatunji, 2012) impact the attention process. For instance, studies have revealed that individuals allocate attention resources to reward-related stimuli (Anderson, 2013). Rewards help stimuli with insignificant features capture attention, even if the rewards subsequently disappear, or the stimulus is independent of the target (Wang, 2016), and when rewards are combined with distraction stimuli, the choice of goals may be hindered (Fan et al., 2014). Numerous studies have found attention biases toward negative stimuli in cognitive processes, which means that individuals detect negative, and threatening stimuli quickly (Jerónimo et al., 2017). Negative faces, especially threatening faces, attract attention, and prolong attention maintenance or reduce attention disengagement ability (Fox et al., 2001). Even if subjects are asked to ignore the emotional information contained in a face, this information still has an impact on the subjects’ responses. Bias toward negative stimuli may occur in one or more phases of attention information processing, involving priming, assessment, or response preparation. However, attention is an important stage of information processing (visual: Deubel et al., 2000; auditory: Näätänen, 1990). The relationship between reward and emotion is likely to appear in the attention stage. Wei et al. (2014) claimed that reward anticipation, which involves waiting and eagerness for upcoming rewards (Oldham et al., 2018) in the reward-appetitive phase (Stavropoulos and Carver, 2014), could promote attention to target-related stimuli or attributes. When an emotional attribute of a stimulus is related to a task, it interacts with reward anticipation, which in turn affects the behavioral response of subjects. Nevertheless, this inference still needs to be supported by empirical research in the field of attention. Eye-tracking technology is commonly used to examine the characteristics of individual attention (Liu and Reichle, 2018; Scholz et al., 2018). An eye tracker can provide continuous dynamic information on subjects during cognitive processing at a high sampling rate; it is more conducive to directly measuring the time course of cognitive processing (especially attention processing) than RTs. Therefore, this study attempts to further address this problem with eye-tracking technology.

Understanding the emotional characteristics of faces is the key to social adaptation and communication skills (Trentacosta and Fine, 2010). In daily life, facial information communicates data on more than one attribute (such as sex, skin color, or expression). When reward anticipation is attached to the emotional and sex attributes of faces, what happens to the attention process? Does the relationship between reward and emotion change depending on the target? This study tried to answer these questions. As mentioned earlier, reward anticipation processing occurs during the appetitive phase of reward processing and has a strong motivational feature that plays an important role in cognitive processes (Yan et al., 2016). Since reward is usually not given in a timely manner under real experimental conditions, they likely reveal anticipation. Previous research has also found that the expectation of reward improves the preparedness of the corresponding brain regions and promotes behavioral responses to subsequent stimulation. Motivational cues bias individuals’ attention resources and target-related information processing by regulating top-down cognitive processes, thereby improving behavioral performance. This study used the cue-target paradigm and eye-tracking technology to examine whether there were differences in behavioral responses and attention characteristics on different targets (emotion recognition and sex recognition) and different reward anticipation (reward and non-reward). Based on previous studies (e.g., Kaltwasser et al., 2013), this experiment predicted that only when the goal was related to the emotion, the reward could promote the processing of emotional information.

## Materials and Methods

### Subjects

Twenty-five students from Zhejiang University of Technology were recruited; 6 of them were excluded because of the low average ratio of valid gaze data, which was less than 70%. Nineteen subjects (9 females, aged 18 to 21 years) had an average ratio of 89.75% for valid gaze data. All subjects had normal or corrected-to-normal visual acuity and were paid with basic rewards. This study was carried out in accordance with the recommendations of the Human Research Ethics Committee of Zhejiang University of Technology. The subjects were recruited through the campus bulletin board and provided signed, informed consent before the experiment. In addition to basic remuneration, the subjects received additional monetary awards based on their experimental performance.

### Design

This study used a 2 × 2 × 2 within-subjects design (target: emotion recognition and sex recognition) × (reward anticipation: non-reward and reward) × (emotional valence: negative and positive). Dependent variables were the subjects’ responses (indexed by RT and accuracy) and attention to the pictures [measured by the first fixation ratio (FFR) and fixation duration ratio (FDR)].

Drawing on the classic paradigm in eye-movement experiments, we simultaneously presented two types of stimuli on one slide to examine attention bias. Positive and negative stimuli, such as high emotional arousal stimuli, would inevitably lead to confusion in the individual emotional experience if presented at the same time. In this study, emotion was an independent variable. Therefore, neutral stimuli were added as controls. The two levels of independent variables, positive and negative emotion, were presented in positive-neutral and negative-neutral pairs.

### Materials

**FIGURE 1:**
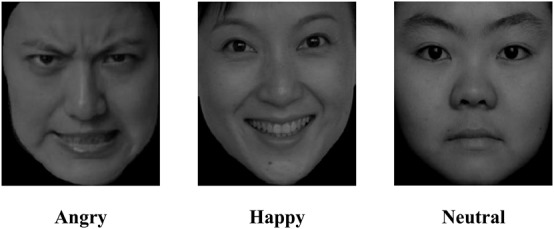
Sample face pictures of different emotional valences.

The experimental materials (sample face pictures of different emotional valences are shown in Figure 1, detail information can be seen in Supplementary Material) included 24 neutral face pictures (calm), 12 positive face pictures (happy), and 12 negative face pictures (angry) (Descriptive statistic of valence, arousal and dominance of material see Table 1), selected from the Chinese Facial Affective Picture System (Wang and Luo, 2005). The sex ratio of each kind of facial expression was 1:1. There was a significant difference between the valences of the three types of face pictures (*ps* < 0.05). Additionally, there was no significant difference between positive and negative faces in arousal or dominance (*ps* > 0.05), while both positive and negative faces had significant differences from neutral faces in arousal and dominance (*ps* < 0.05).

**TABLE 1:** Descriptive statistics of valence, arousal and dominance of material (*M* ± SD).

Emotion	Valence	Arousal	Dominance
Neutral	4.78 ± 0.16	2.55 ± 0.27	2.92 ± 0.28
Positive	7.50 ± 0.17	5.80 ± 0.61	6.50 ± 0.43
Negative	1.97 ± 0.36	6.75 ± 0.76	5.84 ± 0.62

### Instruments

A Tobii X50 telemetry eye tracker with a sampling frequency of 50 Hz was used to track and record the eye movements of the subjects. The program was presented on a 19-inch, 60-Hz cathode ray tube screen. The eye tracker consists of three parts: cameras that take high-resolution images of subjects’ eyes and movement patterns, projectors that create a pattern of near-infrared light on the eye, and algorithms (machine learning, image processing, and mathematical algorithms) that are used to determine the eyes’ position and gaze.

### Procedure

All subjects were instructed to sit approximately 60 cm in front of the display screen and to complete the experiment independently. Based on the requirements of eye-tracking experiments, five-point calibration was used to ensure the accuracy of eye-tracking recording before the experiment started.

**FIGURE 2:**
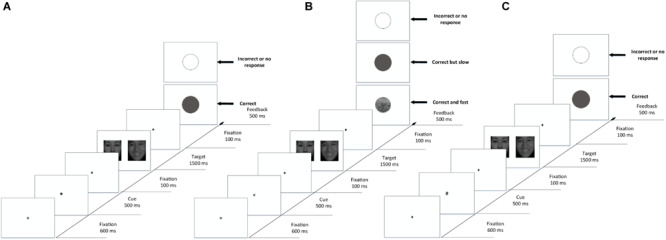
Example displays from the cue-target paradigms used to assess reward-driven attention capture. **(A)** Practice phase with feedback. **(B)** Formal phase with reward feedback. **(C)** Formal phase with non-reward feedback. Each trial was followed by a blank intertrial interval.

The procedure was written using E-prime 2.0. The subjects were asked to complete the emotion recognition task and sex recognition task separately, with corresponding instructions before each task began. The order of the tasks and the correct responses were counterbalanced between the subjects. The cue-target paradigm (Wei et al., 2014) was modified using the experimental procedure illustrated in Figure 2, in which the background was set to white, and the cues and fixations were set to black. Before the experimental procedure began, the subjects were informed of the experimental process and the meaning of the cues and feedback pictures. In the practice phase (shown in Figure 2A), each trial began with the fixation “+” (0.59° × 0.59° visual angle) in the center of the screen for 600 ms. Then, a cue “*” was presented for 500 ms. The fixation appeared again for 100 ms to reset the gaze. After that, two face pictures of different sexes and expressions were presented in pairs. In the emotion recognition task, the subjects were asked to identify the location of pictures according to emotional arousal (high or low). In the sex recognition task, they were asked to identify the location of pictures according to sex (female or male). As one of the objectives in this experiment was to measure the attention maintenance of the subject, the duration of the pictures was fixed to 1500 ms even if the subject responded. Then, the fixation appeared for another 100 ms to reset the gaze, followed by feedback for 500 ms. A gray solid circle appeared on the screen if the subject’s response was correct, and a gray hollow circle appeared on the screen if it was incorrect. Moreover, since the reaction rates had individual differences, the paradigm needed to feed back the RT of subjects to determine whether they would be rewarded. The average RT of every subject in the practice phase was recorded and analyzed as a baseline.

The formal experiment was divided into reward trials (as shown in Figure 2B) and non-reward trials (as shown in Figure 2C). In the reward trials, “¥” was presented as a cue to represent money. The feedback varied depending on the response of the subject: if the subject responded correctly and faster than his or her baseline, a coin would be presented on the screen; if the subject reacted correctly but more slowly than his or her baseline, a gray solid circle appeared on the screen; if the subject reacted incorrectly, a gray hollow circle appeared on the screen. In non-rewarded trials, “#” was presented as a cue to represent no money. The feedback varied depending on the response of the subject: if the subject’s reaction was correct, regardless of how fast the reaction, a gray solid circle appeared on the screen; if the subject’s reaction was incorrect, a gray hollow circle appeared on the screen.

There were 32 trials in the practice phase, of which 16 trials were sex recognition tasks (8 reward trials and 8 non-reward trials), and 16 trials were emotion recognition tasks (8 reward trials and 8 non-reward trials). There were 128 trials in the formal phase, of which 64 trials were sex recognition tasks (32 reward trials and 32 non-reward trials), and 64 trials were emotion recognition tasks (32 reward trials and 32 non-reward trials). In the formal experiment, 8 trials with the same task (recognizing emotion or sex) were used as a block, with a total of 16 blocks. The presentation order of blocks was balanced between subjects. The cues (¥ or #) and the same categories of pictures (such as a happy male face) were presented randomly within blocks.

### Data Analysis

The RTs and accuracies of the subjects in the formal experiment were recorded by E-prime 2.0. E-DataAid was used to collate the data, which were exported to SPSS 22.0 for further statistical analysis. The Bayesian factor (BF_10_) was calculated by JASP^1^ (Wu et al., 2018). JASP provides options for model comparison and data results output. We chose “compare to best model” for model comparison and “across matched models” for calculating the effect of the data. Based on Jeffreys (1961) and Wetzels and Wagenmakers (2012), the interpretation of the Bayesian factor (BF_10_) is presented in Table 2.

**TABLE 2:** Interpretation of Bayesian factors (BF_10_).

Bayesian factor BF_10_	Label
>100	Extremely significant
30–100	Very strongly significant
10–30	Strongly significant
3–10	Moderately significant
1–3	Anecdotally significant
0–1	Not significant

The eye trajectories to faces were determined by presenting positive/negative faces and neutral faces simultaneously as two areas of interest (AOIs) on one slide. The time to first fixation (TFF) and total fixation duration (TFD) data were obtained. TFF is the time point when the gaze of the subject falls on the stimulus for the first time with a latency less than 700 ms and a duration greater than 100 ms. Researchers generally use TFF to reflect subjects’ facilitated attention, which belongs to the automatic processing system and is driven by stimulation. TFF reflects the processing order of a stimulus, which means that the shorter the TFF is, the earlier the AOI is noticed, and the more sensitive or alert an individual is to the stimulus (Cisler and Koster, 2010). TFD is the sum of the fixation durations of subjects to the AOI during the entire stimulus presentation process. Researchers generally use TFD to reflect an individual’s difficulty with disengagement from stimulation (indicating damage to the attention control system) or attention avoidance (reflecting activation of the attention control system), which can reflect the entire cognitive processing of stimulation (Cisler and Koster, 2010).

Time to first fixation and TFD are time variables that are easily affected by individual differences. Differences in results may be caused by differences in the attention features of individuals rather than different experimental conditions. As in previous studies (Wang and Yu, 2017), RT was transformed into the change ratio of RT to exclude the influence of individual differences on the target. We reanalyze TFF and TFD in the form of ratios to define FFR and FDR as new dependent variables. FFR is defined as the ratio of the number of trials with a quicker TFF in a positive/negative AOI to the total number of trials for the same experimental condition compared with a neutral AOI. For example, there were 32 negative-neutral reward trials in the emotion recognition task. If there were 24 trials with a quicker TFF for negative faces than for neutral faces, then the FFR in negative-neutral reward trials would be 0.75. The calculation of FDR is also based on two AOIs of positive/negative and neutral faces. FDR is defined as the ratio of the total TFD in a positive/negative AOI to the sum of TFDs in two AOIs under the same experimental conditions. For example, if the total TFD of negative faces were 16000 ms and the sum of the TFDs of negative and neutral pictures were 20000 ms in the 32 negative-neutral reward trials in the emotion recognition task, the FDR would be 0.8.

The statistical analyses we used are presented as follows. First, accuracy and RT were analyzed separately using repeated-measures ANOVA, taking target (emotion recognition and sex recognition), reward anticipation (reward and non-reward), and emotional valence (negative and positive) as factors. Regarding the target differences, we performed a separate repeated-measures ANOVA for each task with reward anticipation and emotional valence as factors. Since neutral faces and emotional faces were presented at the same time, we integrated FFR and FDR into the positive condition and negative condition, taking 0.5 as the expected value to perform a one-sample *t*-test. Finally, FFR and FDR as eye-tracking indexes were separately analyzed using repeated-measures ANOVA, taking reward anticipation, emotional valence, and target as factors. We also performed a separate repeated-measures ANOVA for each target with experimental reward anticipation and emotional valence as factors. Where a significant difference was found between factors, Student’s *t*-test was used.

## Results

### Behavioral Data

ANOVA results (descriptive statistics are reported in Table 3) show that the interaction between target and emotion was significant in terms of accuracy [*F*(1,18) = 17.15, *p* = 0.001, η*_*p*_^2^* = 0.49, and *BF*_*10*_ = 1083.61] and RT [*F*(1,18) = 4.97, *p* < 0.05, η*_*p*_^2^* = 0.22, *BF*_*10*_ = 1.24]. The simple effect test found that in the sex recognition task, there was a higher accuracy (*p* < 0.05) and a shorter RT (*p* < 0.05) under the positive condition, indicating superior processing toward positive faces, and that there was no significant difference in the emotion recognition task. The main effect of emotion on accuracy was significant [*F*(1,18) = 8.75, *p* < 0.05, η*_*p*_^2^* = 0.33, and *BF*_*10*_ = 16.46], reflecting that accuracy under the positive condition was higher than that under the negative condition. Other main effects and interactions were not significant (*ps* > 0.05). Separate repeated-measures ANOVA results showed that in the emotion recognition task, the main effect of reward anticipation was significant [*F*(1,18) = 5.18, *p* < 0.05, η*_*p*_^2^* = 0.22, and *BF*_*10*_ = 2.67]. This result reflects a shorter RT under the reward condition than under the non-reward condition and indicates a behavior bias toward reward. In the sex recognition task, the main effect of emotion was significant [*F*(1,18) = 4.50, *p* < 0.05, η*_*p*_^2^* = 0.20, and *BF*_*10*_ = 1.01], manifesting as a shorter RT under the positive condition than under the negative condition. Other main effects and interactions were not significant (*ps* > 0.05).

**TABLE 3:** Descriptive statistics of accuracy and RT under different conditions (*M* ± SD).

		Accuracy		RT (ms)	
Target	Reward anticipation	Positive	Negative	Positive	Negative
Emotion	Reward	0.92 ± 0.07	0.93 ± 0.07	740.25 ± 156.85	729.19 ± 131.93
	Non-reward	0.94 ± 0.06	0.95 ± 0.05	784.56 ± 161.5	762.14 ± 127.50
Sex	Reward	0.98 ± 0.03	0.91 ± 0.08	759.47 ± 138.40	791.69 ± 153.80
	Non-reward	0.98 ± 0.03	0.91 ± 0.05	762.65 ± 118.85	794.42 ± 157.79

### Eye-Tracking Data

**FIGURE 3:**
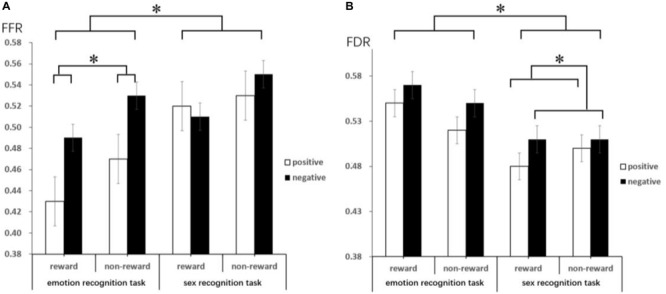
This figure reveals the mean FFR **(A)** and FDR **(B)** by target, emotion type and reward anticipation. Error bars represent the standard error of the mean. These lines mainly indicate that there are significant differences under different conditions through the difference comparison test. ^∗^Represents *p* < 0.05.

The results of the one-sample *t*-test (descriptive statistics are reported in Table 4 and Figure 3) show that FFR was marginally significantly different under negative conditions [*t*(18) = 2.06, *p* = 0.06, *d* = 0.67, and *BF*_*10*_ = 2.55], indicating an attention bias toward negative faces. FDR was also significantly different under negative conditions [*t*(18) = 2.58, *p* < 0.05, *d* = 0.84, and *BF*_*10*_ = 6.13], suggesting that negative faces were able to hold attention longer than neutral faces. Under positive conditions, comparing FFR and FDR with 0.5, no significant difference could be asserted (*ps* > 0.05).

**TABLE 4:** Descriptive statistics of FFR and FDR under different conditions (*M* ± SD).

		FFR		FDR	
Target	Reward anticipation	Positive	Negative	Positive	Negative
Emotion	Reward	0.43 ± 0.10	0.49 ± 0.03	0.55 ± 0.13	0.57 ± 0.14
	Non-reward	0.47 ± 0.07	0.53 ± 0.11	0.52 ± 0.10	0.55 ± 0.10
Sex	Reward	0.52 ± 0.11	0.51 ± 0.13	0.48 ± 0.19	0.51 ± 0.05
	Non-reward	0.53 ± 0.10	0.55 ± 0.12	0.50 ± 0.05	0.51 ± 0.06

The ANOVA results show that the main effect of the target was significant [*F*(1,18) = 18.13, *p* = 0.000, η*_*p*_^2^* = 0.50; and *BF*_*10*_ = 10.02] on FFR, with a lower FFR in the emotion recognition task than in the sex recognition task. The main effect of emotion was also significant [*F*(1,18) = 5.82, *p* = 0.03, η*_*p*_^2^* = 0.24; and *BF*_*10*_ = 1.03]; the FFR of positive faces was lower than that of negative faces, which also indicated a negative bias. Other main effects and interactions were not significant (*ps* > 0.05). Separate repeated-measures ANOVA results show that in the emotion recognition task, the main effect of reward anticipation was significant [*F*(1,18) = 5.37, *p* = 0.03, η*_*p*_^2^* = 0.23; and *BF*_*10*_ = 1.89], with a significantly lower FFR under the reward condition than under the non-reward condition. Additionally, the main effect of emotion was significant [*F*(1,18) = 6.88, *p* = 0.02, η*_*p*_^2^* = 0.28; and *BF*_*10*_ = 13.40], with a lower FFR for positive faces than for negative faces, while the interaction between emotion and reward anticipation was not significant. In the sex recognition task, other main effects and interactions were not significant (*ps* > 0.05).

The ANOVA results indicate that the main effect of the target was marginally significant [*F*(1,18) = 4.08, *p* = 0.059, η*_*p*_^2^* = 0.19; and *BF*_*10*_ = 246.22] on FDR, with a higher FDR in the emotion recognition task than in the sex recognition task. The main effect of emotion was also significant [*F*(1,18) = 6.67, *p* < 0.05, η*_*p*_^2^* = 0.27; and *BF*_*10*_ = 1.07], with a lower FDR for positive faces than for negative faces. Other main effects and interactions were not significant (*ps* > 0.05). Separate repeated-measures ANOVA results show that in the sex recognition task, the main effect of emotion was significant [*F*(1,18) = 4.41, *p* < 0.05, η*_*p*_^2^* = 0.20; and *BF*_*10*_ = 4.07], with a lower FDR for positive faces than for negative faces. Other main effects and interactions were not significant (*ps* > 0.05).

## Discussion

No interaction between reward anticipation and emotion was found in the emotion recognition or sex recognition tasks in this study. However, we found that the results in the two tasks were completely different. When the target was related to emotion (emotion recognition task), consistent with the research by Wei et al. (2014), the main effect of reward anticipation was significant. This may be because reward anticipation promotes the processing of the emotional attributes of a stimulus, so in the reward trials, the subjects showed shorter RTs and higher FFRs. While the target was unrelated to emotion (sex recognition task), consistent with the research by Kaltwasser et al. (2013), the main effect of reward anticipation was not significant. The subjects focused on the sex information of the stimulus, which interfered with the automatic processing of the emotional attributes of the stimulus. The processing of the emotional attributes of a stimulus is superior to the processing of other attributes to a certain extent, and it has a certain impact on the processing of other attributes (Yang et al., 2016); thus, reward anticipation promotes the processing of emotion and sex information at the same time. In a word, it suggests that the target itself regulates the effect that reward anticipation work on the processing target attribute. According to the theory of Murray (2007), reward anticipation cues influence behavior, whose underlying mechanism comprises two systems inside the amygdala running in parallel. One system can adjust the universal arousing effect of reward anticipation, while the other links the sensory properties of reward anticipation with emotion. Therefore, in the emotion recognition task, the two systems work together, resulting in shorter RTs and higher FFRs in reward trials. Meanwhile, the two systems of reward anticipation inside the amygdala run separately, resulting in the effect of reward anticipation being dispersed, which causes no significant main effect of reward anticipation. In other words, reward anticipation promotes emotion processing explicitly and automatically, but the processing of emotional information (such as the emotional attributes of stimuli) disperses part of the promotion effect of reward anticipation. The processing of emotion stimuli increases processing speed only when it is rewarded.

Consistent with previous studies (Hartikainen et al., 2014), this study indicated that subjects had a negative emotional bias during the face recognition process. Individuals exhibited a priority effect on unpleasant stimuli, especially threatening stimuli such as violence, bloody scenes and angry faces, which affected psychological processes and behavioral responses (Buckner et al., 2010). Similarly, the target influenced the effect of emotion on the response of the emotion process. According to the perceptual load theory, when attention resources are completely occupied by task-related content, the process of dealing with task-free interference will stop (Neumann et al., 2011), and emotional bias is affected by the perceptual load (Luo et al., 2017). Negative emotional bias appeared in the sex recognition task, indicating that some attention resources were allocated to emotional processing. However, in the emotion recognition task, compared with positive faces, subjects gazed at negative faces for a longer time but with lower processing quality. This was likely because more attentional resources were used to alert individuals to negative stimuli, and fewer cognitive resources were used for target-related processing, resulting in a higher RT (Zhu and Zhu, 2011; Ji, 2013) or lower accuracy. This indicates that the emotional valence of the stimulus might affect the overall attention processing quality. Individuals have a need to stay in a neutral state, and they may need to spend additional resources regulating the effect of negative emotion when processing stimuli. This reduces the processing speed and interference accuracy of the target-related process.

Although the target did not impact the interaction between reward anticipation and emotion, the results suggested that the main effect of the target was significant for FFR and FDR, which indicated that the target might affect the emotion attention process independently. Compared with the sex recognition task, the subjects had lower FFRs and higher FDRs in the emotion recognition task. This meant that the individuals tried to avoid emotional faces while holding a high level of attention maintenance when completing an emotion-related task. According to previous studies, if the target is valence related, the valence of faces will have additional effects on attention resource allocation (Schulz et al., 2013).

The rapid and effective identification and analysis of various types of information in complex environments are of great significance to the adaptation and development of individuals. The results of this study provide theoretical support for understanding individuals’ emotion processing. This study found that reward anticipation promotes emotion processing explicitly and implicitly. We required the subjects to respond as quickly as possible during the practice trials (baseline). And in the formal trials, subjects can receive rewards only when their response was faster than the baseline, which required the subjects to pay close attention to the target. Besides, implicit processing of emotional faces included recognition of other facial cues, such as sex (Scheuerecker et al., 2007). In the emotion recognition task, in which emotion processing is explicit processing of faces, consistent with the research by Wei et al. (2014), the results show that reward anticipation promotes emotion processing; that is, reward anticipation promotes explicit emotion processing. In the sex recognition task, emotion processing is implicit processing of faces. The subjects showed higher processing quality in a shorter fixation duration for positive faces, while reward anticipation did not promote sex processing. It is inferred that this was due to the emotional content being automatically processed and interfered the effect of reward anticipation on sex processing (Rigoulot et al., 2012), and emotion processing was promoted, so we speculate reward anticipation promoted implicit emotion processing.

## Conclusion

This study, which adopted a cue-target paradigm to explore the role of the target in the relationship between reward anticipation and emotion, drew the following conclusions: Target status can regulate the promotion of reward anticipation to emotional attention. Reward anticipation promotes explicit emotion processing. The emotional relevance of the target can impact the orientation and maintenance of attention to emotional faces. Emotional attributes may take processing priority over other attributes to a certain extent.

## Data Availability Statement

The database generated for this study is available upon request to the corresponding author.

## Ethics Statement

This study was carried out in accordance with the recommendations of The ZJUT Human Research Ethics Committee. All subjects gave written informed consent in accordance with the Declaration of Helsinki. The protocol was approved by The ZJUT Human Research Ethics Committee.

## Author Contributions

YY and YX developed the study concept and design. Data collection was performed by RW. YY and YX performed the data analysis. All authors contributed to the data interpretation and manuscript writing and approved the final version of the manuscript for submission.

## Conflict of Interest

The authors declare that the research was conducted in the absence of any commercial or financial relationships that could be construed as a potential conflict of interest.

## Footnotes

1 https://jasp-stats.org/
