# Supplementary figures and images for: Regulatory Effects of Reward Anticipation and Target on Attention Processing of Emotional Stimulation
Source: Front Psychol. 2020 Jun 3;11:1170. doi: 10.3389/fpsyg.2020.01170 (PMC7283617; doi:10.3389/fpsyg.2020.01170)

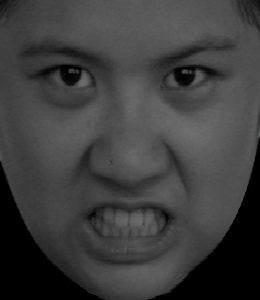

Supplement: Supplementary file 1 [file Image_1.PNG]

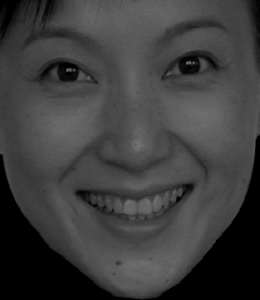

Supplement: Supplementary file 2 [file Image_2.PNG]

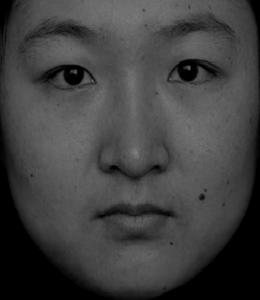

Supplement: Supplementary file 3 [file Image_3.PNG]
